# Supplementary material for: The effect of the COVID-19 pandemic on health care workers’ anxiety levels: a meta-analysis
Source: PeerJ. 2022 Apr 11;10:e13225. doi: 10.7717/peerj.13225 (PMC9009329; doi:10.7717/peerj.13225)
Supplement: Supplemental Information 1 [file peerj-10-13225-s001.doc]

In the COVID-19 pandemic, Health care workers have to face hitherto unknown challenges in both physical and mental health causes excessive tension and anxiety in health care workers. While anxiety is a common mental condition that can results in psychological distress and even affects the daily lives of individuals. Anxiety also impairs the executive functions that underlie our ability to control and focus on our thoughts. Consequently, studying and accurately grasping the anxiety levels of health care workers is necessary to take more appropriate and corrective measures to deal with public health and safety and we think meta-analysis is the most appropriate approach.

Although some researchers have investigated health care workers’ anxiety levels during the COVID-19 pandemic, many new papers on COVID-19 were being released rapidly since the pandemic still poses a serious threat. The present meta-analytic study included the latest papers, and aimed to generate a more comprehensive understanding of the prevalence of anxiety among health care workers. Furthermore, We consider it necessary to compare health care workers’ anxiety levels in COVID-19 with anxiety levels before COVID-19 to find out whether the COVID-19 pandemic really increased health care workers’ anxiety level.
